# Routine Pringle Maneuver During Recipient Hepatectomy Is Associated with Improved Perioperative and Early Graft Outcomes After Adult Liver Transplantation

**DOI:** 10.3390/jcm15207939

**Published:** 2026-10-14

**Authors:** Ashraf Imam, Ahmad Salhab, Suha Shabaneh, Michael Rivin, Alaa Altiti, Mohammad Emar, Natalia Kuzmina, Penina Yehudit Dienstag, Rifaat Safadi, Abed Khalaileh

**Affiliations:** 1 Transplantation Unit, Department of Surgery, Hadassah Medical Organization, Hadassah-Hebrew University, Jerusalem 9112001, Israel; ahmad.salhab@mail.huji.ac.il (A.S.); suhasuha1987@gmail.com (S.S.); rivin@hadassah.org.il (M.R.); altitia@hadassah.org.il (A.A.); memar@hadassah.org.il (M.E.); 2 Department of Anesthesiology, Hadassah Medical Organization, Hadassah-Hebrew University, Jerusalem 9112001, Israel; nataliaku@hadassah.org.il (N.K.); pdienstag@hadassah.org.il (P.Y.D.); 3 Liver Institute, Hadassah Medical Organization, Hadassah-Hebrew University, Jerusalem 9112001, Israel; safadi@hadassah.org.il

**Keywords:** liver transplantation, pringle maneuver, recipient hepatectomy, early allograft dysfunction, perioperative bleeding

## Abstract

**Background:** Recipient hepatectomy during orthotopic liver transplantation (OLT) is frequently complicated by severe bleeding due to portal hypertension and collateral circulation. The Pringle maneuver’s benefit in this setting remains uncertain. **Methods:** We retrospectively analyzed data from 141 adults who underwent primary deceased-donor OLT at a single center between January 2020 and June 2026. A routine intermittent Pringle maneuver was introduced in 2023; 79 subsequent recipients comprised the Pringle group, while 62 earlier recipients served as historical controls. Multivariable regression and inverse probability of treatment weighting (IPTW) were used to address confounding, and temporal trends within each period were examined. **Results:** Indication for transplantation, markers of hepatic decompensation, and extended-criteria donor status did not differ between groups. Compared with controls, the Pringle group required fewer packed red blood cell units (median 2 {IQR 0–4} vs. 3 {1–5}; *p* = 0.046), fresh frozen plasma units (4 {0–6} vs. 4 {3–8}; mean 4.1 vs. 6.9; no plasma transfused in 30.8% vs. 13.6%; *p* = 0.005), and total blood products (5 {0–9} vs. 8 {4–14}; *p* = 0.009). Recipient hepatectomy was shorter (129 {105–165} vs. 177 {146–208} minutes; *p* < 0.001), and postoperative bleeding (2.5% vs. 22.6%; *p* < 0.001), biliary leak (2.5% vs. 11.3%; *p* = 0.043), and early allograft dysfunction (EAD; 6.3% vs. 29.0%; *p* < 0.001) were less frequent. After adjustment, the Pringle maneuver remained associated with shorter hepatectomy (time ratio 0.76; 95% CI 0.67–0.87), reduced fresh frozen plasma (IRR 0.60; 0.40–0.91) and total blood product use (IRR 0.61; 0.40–0.92), and lower odds of postoperative bleeding (OR 0.04; 0.01–0.25), biliary leak (OR 0.13; 0.02–0.63), and EAD (OR 0.14; 0.04–0.58). Packed red blood cell transfusion was nonsignificant after adjustment (IRR 0.70; 0.43–1.12). IPTW yielded consistent findings. Overall survival did not differ (*p* = 0.885). **Conclusions:** In this historical-control study, routine intermittent Pringle maneuver was associated with improved operative efficiency, lower transfusion requirements, and fewer early complications without affecting survival; given the retrospective design and the modest absolute differences in transfusion, these findings require prospective confirmation.

## 1. Introduction

Orthotopic liver transplantation (OLT) is a highly effective treatment for those suffering not only from end-stage liver disease, but also from select primary liver cancers [1,2,3]. While surgical techniques, organ preservation, and postoperative care and medication have improved tremendously, the surgical removal of the recipient’s liver is one of the most challenging and continues to be a major contributor to intraoperative blood loss [4,5,6]. Severe portal hypertension, extensive collateral circulation, and dense adhesions of the liver, as well as a coagulopathic state, impede the surgical removal of the native liver [7,8]. As a result, the need for blood transfusions increases, and the duration of the operation is extended. Moreover, there is a marked increase in postoperative morbidity and early allograft dysfunction, as well as a decrease in graft and long-term patient survival, after blood transfusions in the perioperative period. This emphasizes the necessity for techniques to decrease blood loss during recipient hepatectomy [9,10,11].

The Pringle maneuver is a method that achieves temporary control of hepatic inflow and is used in liver resection [12,13,14,15,16]. It temporarily ceases blood flow to the liver and transiently interrupts the portal circulation, thereby decreasing blood loss, improving surgical exposure, and facilitating liver dissection [17]. Current reviews and analyses of many studies have shown that the Pringle maneuver actually decreases blood loss and the need for blood transfusions in liver resection and does not increase postoperative morbidity when it is used appropriately [18,19,20]. However, the context of a liver resection is different from the context of a recipient liver removal in liver transplantation surgery. Severe cirrhosis, portal hypertension, compromise of the systemic circulation, and the transplant liver’s own ischemia–reperfusion injury are unique to liver transplantation [21]. Therefore, the use of the Pringle maneuver in liver transplantation surgery cannot be assumed to have the same effects as when it is used in liver resection.

Investigating the Pringle maneuver in liver transplantation remains problematic for various reasons. Reports primarily constitute retrospective case series focusing on a handful of patients, and the small amount of prospective literature addresses only living-donor liver transplantation [22,23,24]. Additionally, the literature primarily examines blood loss and the need for blood product transfusions, while complications of clinical relevance, including postoperative bleeding, biliary complications, early allograft dysfunction, and patient and allograft survival, have not been thoroughly examined [25,26]. Therefore, it remains uncertain whether the routine application of hepatic inflow occlusion in recipient hepatectomy optimizes the procedure and provides additional perioperative and early graft-function benefits beyond the reduction in intraoperative bleeding.

Starting in 2023, our institution implemented the routine use of the Pringle maneuver in recipient hepatectomy for adult OLT and provided the opportunity to assess the clinical effect of this practice in a current transplant population. Thus, the objectives of this study were to determine the effects of routine occlusion of hepatic inflow on adult liver transplantation and to assess whether the maneuver decreased the need for transfusions, improved surgical efficiency, decreased postoperative complications, and improved early graft function. We believed the routine use of the Pringle maneuver would reduce perioperative bleeding, improve early transplant outcomes, and would not adversely impact graft function.

## 2. Materials and Methods

### 2.1. Study Design and Patient Population

This was a retrospective single-center cohort study of prospectively collected data that included adult patients who underwent orthotopic liver transplantation at Hadassah Medical Center, Jerusalem, Israel, between January 2020 and June 2026. In the study period, the Pringle maneuver was implemented routinely and intermittently during recipient hepatectomy as part of the surgical practice starting January 2023. Therefore, patients who underwent transplantation before January 2023 comprised the No-Pringle group, and those who underwent transplantation after January 2023 comprised the Pringle group. During the study period, all transplantations were performed by the same two senior attending transplant surgeons, both with extensive experience in liver transplantation, and no other change in the operative technique, anesthetic protocol, or immunosuppression regimen was introduced during the study period, apart from the use of an internal biliary stent in some recipients of the No-Pringle group, as described below. To reduce variability in the surgical procedure, other organ transplantation cases, combined liver-kidney transplantation, and retransplantation cases were excluded. After excluding 15 retransplantations, a total of 141 adult primary liver transplantations were included in the study, of which 79 were in the Pringle group and 62 in the No-Pringle group. The study received approval from the institutional review board of Hadassah Medical Center HMO-0453-24 and was conducted in accordance with the ethical principles of the Declaration of Helsinki. Because the study was retrospective, formal consent was waived.

### 2.2. Surgical Technique

All transplantations were performed by the same two experienced transplant surgeons using standard orthotopic liver transplantation techniques; we usually approach the left and right lateral attachments of the liver until exposing the vena cava, then dissection of the porta hepatis is performed, and then dissection and closure of the short hepatic veins, followed by closure of the right hepatic vein, is performed. Then, stapling of the portal vein, followed by the middle and left hepatic veins, is performed to complete the hepatectomy phase while preserving the vena cava. Beginning in January 2023, intermittent hepatic inflow occlusion was routinely performed during recipient hepatectomy by temporary clamping of the hepatoduodenal ligament (Pringle maneuver) using a 14 French Foley catheter. The maneuver was applied intermittently, typically in two to three cycles (median, two cycles), with each period of inflow occlusion lasting approximately 10–15 min, followed by a 5 min reperfusion interval. The number and duration of clamping cycles were determined intraoperatively according to the patient’s hemodynamic status and the presence of signs of small bowel venous engorgement. The maneuver was applied intermittently according to intraoperative surgical judgment to facilitate recipient hepatectomy and improve operative visualization while minimizing blood loss. Before 2023, recipient hepatectomy was performed without routine hepatic inflow occlusion. Recipient hepatectomy was performed with preservation of the retrohepatic inferior vena cava in both periods, using a side-to-side cavo-caval anastomosis and without a temporary portocaval shunt or venovenous bypass, and the sequence of dissection, the technique of graft implantation, and the technique of biliary reconstruction (duct-to-duct anastomosis with a running suture whenever the recipient duct was of adequate caliber and quality, or Roux-en-Y hepaticojejunostomy when duct-to-duct anastomosis was contraindicated) did not change between the two periods, except that an internal plastic stent was placed across the duct-to-duct anastomosis in some recipients of the No-Pringle group; stents were not used in the Pringle group. Graft implantation time corresponds to the warm ischemia time, defined as the interval from placement of the graft in the abdomen to venous reperfusion; this variable is reported in Table 1 and was included in the adjusted analyses. The end of surgery was also recorded; however, total operative time was not considered a reliable measure of surgical difficulty, because in a large number of cases the team took a pause of 15 to 45 min after arterial reperfusion to achieve hemostasis. Recipient hepatectomy duration was therefore used as the principal operative-time variable.

### 2.3. Donor and Graft Characteristics

All grafts were whole livers procured from donation after brain death (DBD) donors; donation after circulatory death is not practiced in Israel, and no living-donor or split grafts were included. Grafts were preserved by static cold storage. Ex situ machine perfusion was used for a single graft in the Pringle group (2026), and this recipient was retained in the analysis. Extended-criteria donor status was recorded prospectively in the institutional transplant database according to the following institutional criteria: donor age > 65 years, macrovesicular steatosis > 30%, cold ischemia time > 12 h, hypernatremia, or prolonged intensive care unit stay. Pre-implantation graft biopsy was not performed routinely; it was obtained only when indicated, namely when steatosis was suspected on inspection or for extended-criteria donors; because histological grading of graft steatosis and fibrosis was not systematically recorded, these parameters could not be analyzed.

### 2.4. Anesthetic Management and Transfusion Practice

Anesthetic management was provided by the institutional liver transplant anesthesia team according to a standard protocol that did not change during the study period. Intraoperative coagulation was monitored mainly by clinical assessment and conventional laboratory tests (prothrombin time/INR, activated partial thromboplastin time, and platelet count); thromboelastography (TEG) was available but was used only occasionally, at the discretion of the anesthesiologist, and was not part of routine practice during the study period. PRBCs were transfused for a hemoglobin concentration below 7 g/dL before surgery and, intraoperatively, according to hemodynamic status and the extent of surgical bleeding, and FFP was administered to correct the coagulation profile (INR above 2.1) and whenever the surgeon reported diffuse non-surgical bleeding suggestive of coagulopathy. Platelet concentrates were transfused for a platelet count below 50,000/µL, and cryoprecipitate was administered at the discretion of the attending anesthesiologist according to the platelet count and INR. Because the transplant database did not capture hemostatic products other than PRBC and FFP, intravenous fluid volumes, vasopressor or inotrope doses, or hemodynamic changes after graft reperfusion (post-reperfusion syndrome), these variables could not be analyzed.

### 2.5. Data Collection

Demographic information about transplantation recipients and organ donors, several key variables, and clinical outcomes were retrieved from both the institutional liver transplantation database and electronic medical records. Recipient information included age, sex, BMI, physiological MELD score (calculated from the values obtained immediately before transplantation, reflecting disease severity at the time of surgery), the primary indication for transplantation, the presence of ascites and hepatic encephalopathy, and the location of the patient immediately before transplantation (home, hospital ward, or intensive care unit). Donor information was limited to age, extended-criteria status, and ischemia times. The primary indication for transplantation was classified from the clinical record as metabolic dysfunction-associated steatotic liver disease (MASLD), viral hepatitis (hepatitis B or C virus), autoimmune or cholestatic liver disease, alcohol-related liver disease, or other; hepatocellular carcinoma (HCC) was recorded separately, irrespective of the underlying etiology. Only previous upper abdominal surgery was documented, and it did not differ between the two groups. Hepatectomy duration, packed red blood cells (PRBCs), fresh frozen plasma (FFP), and complications were included as operative variables. Post-transplantation mortality and retransplantation were verified using data from the Israeli National Transplant Registry to ensure data accuracy.

### 2.6. Study Outcomes

The primary outcome was intraoperative PRBC transfusion requirement. Because direct measurement of intraoperative blood loss was not routinely available, PRBC transfusion was used as a surrogate marker of clinically significant intraoperative bleeding. Secondary outcomes included FFP transfusion, recipient hepatectomy duration, postoperative bleeding, biliary leak, early allograft dysfunction (EAD), and overall survival. Total intraoperative blood product transfusion, defined for each recipient as the sum of PRBC and FFP units administered during the transplant procedure, was additionally analyzed as a composite measure of overall transfusion burden. The duration of the recipient hepatectomy was defined as the time from the start of surgery to liver extraction.

Early allograft dysfunction was defined by the criteria of Olthoff et al. as the presence of at least one of the following: bilirubin level of at least 10 mg/dL on postoperative day 7, an INR level of at least 1.6 on postoperative day 7, or an elevated serum AST or ALT of greater than 2000 IU/L in the first postoperative week.

Postoperative bleeding and a biliary leak were observed as binary clinical outcomes. Total survival was defined as the period from transplant to death or the last date of follow-up, which was July 2026.

### 2.7. Statistical Analysis

Distributions of continuous variables were evaluated and reported as medians (IQRs). Study groups were compared using the Mann–Whitney U test. The Mann–Whitney U test compares the entire distributions of the two groups rather than their medians; where medians were identical, transfusion requirements were additionally summarized by the mean, the proportion of recipients requiring no transfusion, the 75th and 90th percentiles, and the Hodges–Lehmann estimate of the difference with a bootstrap 95% confidence interval. For categorical variables, frequencies and percentages were calculated. Group comparison was performed using the χ^2^ or Fisher’s exact test as appropriate. Percentages were calculated among recipients with available data for each variable.

To address potential baseline differences among the two eras, separate multivariable regression models were constructed for each outcome. Recipient age, physiological MELD score, donor age, and ischemia time were included as covariates. Warm ischemia time was included in all models except the EAD analysis, in which cold ischemia time was included because of its significant association with graft dysfunction.

Recipient hepatectomy duration was modeled using log-transformed linear regression, and results were reported as ratios for duration. PRBC, FFP, and total blood product transfusion requirements were modeled using negative binomial regression, and results were reported as incidence rate ratios (IRR). Other outcomes were modeled using logistic regression, and results were reported as odds ratios (OR). Because the number of biliary leak events was small (n = 9), the corresponding logistic model was fitted by Firth’s penalized maximum likelihood, in which the likelihood is penalized by the Jeffreys invariant prior to reduce small-sample bias and to guarantee finite estimates when events are sparse. For this model, the 95% confidence interval and *p*-value were derived from the penalized profile likelihood rather than from Wald statistics, because Wald-based intervals are unreliable at low event counts. All other binary outcomes were fitted by ordinary maximum-likelihood logistic regression. Mortality was assessed using Cox proportional hazards regression and reported as hazard ratios (HR).

Propensity scores, using the covariates recipient age and sex, BMI, physiological MELD score, donor age, and warm ischemia time, were estimated with logistic regression. To further reduce confounding, inverse probability of treatment was used to weight outcome models. Before and after weighting, the balance of covariates was assessed using standardized mean differences. Overall survival was assessed with the Kaplan–Meier method and compared using the log-rank test.

To examine whether the observed differences could reflect a gradual gain in institutional experience rather than the introduction of the Pringle maneuver, temporal trends in recipient hepatectomy duration and transfusion requirements were examined within each study period by Spearman rank correlation with the date of transplantation and by comparing the first and second halves of each period with the Mann–Whitney U test. Baseline variables reported for the first time in this revision (indication for transplantation, HCC, ascites, hepatic encephalopathy, pre-transplant location, and extended-criteria donor status) were compared descriptively between groups; because none differed significantly, the prespecified covariate sets of the multivariable and propensity-score models were retained.

Statistical analyses were performed using Python 3.12 (pandas, SciPy, statsmodels, and lifelines packages). The Firth penalized-likelihood model was implemented directly following Heinze and Schemper.

## 3. Results

### 3.1. Baseline Characteristics of the Study Cohort

A total of 141 adult recipients undergoing primary orthotopic liver transplantation were included in the final analysis, comprising 79 patients in the Pringle group and 62 patients in the No-Pringle group. Baseline recipient and donor characteristics are summarized in Table 1. Recipient age was comparable between groups (median 62 {IQR, 53–68} vs. 58 {46–66} years, *p* = 0.055), as were sex distribution (59.5% vs. 62.9% male, *p* = 0.812), body mass index (26.9 {24.2–31.4} vs. 26.8 {24.0–31.}] kg/m^2^, *p* = 0.958), physiological MELD score (26 {23–31} vs. 24 {22–27}, *p* = 0.065), and cold ischemia time (303 {260–352} vs. 315 {262–350} minutes, *p* = 0.587).

The primary indication for transplantation did not differ between groups (*p* = 0.542). MASLD was the leading indication in both groups (43.4% vs. 36.2%), followed by autoimmune or cholestatic liver disease (11.8% vs. 20.7%), viral hepatitis (11.8% vs. 12.1%), and alcohol-related liver disease (7.9% vs. 3.4%); the remaining recipients were transplanted for acute or acute-on-chronic liver failure, inherited metabolic disease, Budd–Chiari syndrome, or cryptogenic and other causes. HCC was present in 30.3% and 19.0% of recipients, respectively (*p* = 0.197). Markers of hepatic decompensation were similar or numerically more frequent in the Pringle group, including ascites (60.3% vs. 54.2%, *p* = 0.595), hepatic encephalopathy of any grade (47.4% vs. 30.5%, *p* = 0.068), and transplantation directly from the intensive care unit (16.7% vs. 8.5%; *p* = 0.200 for the overall comparison of pre-transplant location). The proportion of grafts from extended-criteria donors was 50.0% in the Pringle group and 61.0% in the No-Pringle group (*p* = 0.267).

Two baseline variables differed significantly between groups. Donor grafts were younger in the Pringle group than in the No-Pringle group (53 {40–71} vs. 65 {56–74} years, *p* = 0.007), and warm ischemia time was modestly shorter (33 {29–38} vs. 35 {31–40} minutes, *p* = 0.028). These variables were considered clinically relevant potential confounders because both donor age and warm ischemia time are known determinants of early graft function. Accordingly, they were incorporated into the multivariable regression and propensity-weighted analyses.

### 3.2. Perioperative and Early Postoperative Outcomes

Perioperative and early postoperative outcomes are summarized in Table 2, and the principal perioperative outcomes are presented in Figure 1 and Figure 2. Routine application of the Pringle maneuver was associated with significantly lower intraoperative transfusion requirements and shorter recipient hepatectomy. Recipients in the Pringle group received fewer PRBC units than those in the No-Pringle group (median 2 {IQR, 0–4} vs. 3 {1–5} units, *p* = 0.046), together with lower FFP transfusion requirements (median 4 {0–6} vs. 4 [3,4,5,6,7,8] units, *p* = 0.005). Although the median FFP requirement was identical in the two groups, the distributions differed: 30.8% of Pringle-group recipients received no FFP compared with 13.6% of controls, the mean requirement was 4.1 vs. 6.9 units, the 75th and 90th percentiles were 6 and 8 vs. 8.5 and 15 units, and the Hodges–Lehmann estimate of the difference was −2 units (95% CI, −4 to 0). The Mann–Whitney U test compares the two distributions as a whole rather than their medians; the probability that a randomly selected Pringle-group recipient received fewer FFP units than a randomly selected control was 0.64. The full distributions of FFP transfusion in the two groups are shown in Supplementary Figure S2. Considering all blood products together, the total intraoperative transfusion requirement was likewise lower in the Pringle group (median 5 {IQR, 0–9} vs. 8 {4–14} units, *p* = 0.009), and a greater proportion of Pringle-group recipients required no intraoperative transfusion at all (25.3% vs. 9.7%, *p* = 0.031). Recipient hepatectomy duration was also significantly shorter in the Pringle group (129 {105–165} vs. 177 {146–208} minutes, *p* < 0.001).

Significant differences were observed across several early postoperative outcomes (Table 2). Most notably, EAD occurred in only 5 of 79 recipients (6.3%) in the Pringle group compared with 18 of 62 recipients (29.0%) in the No-Pringle group (*p* < 0.001). In addition, postoperative bleeding was substantially less frequent following application of the Pringle maneuver (2.5% vs. 22.6%, *p* < 0.001), while biliary leakage was also significantly reduced (2.5% vs. 11.3%, *p* = 0.043). In contrast, no significant differences were observed for the remaining postoperative outcomes. The incidence of postoperative ileus was low in both groups (1.3% vs. 1.6%, *p* = 1.000). Similarly, overall mortality during follow-up did not differ significantly between the Pringle and No-Pringle groups (12.7% vs. 16.1%, *p* = 0.731).

### 3.3. Adjusted and Propensity-Weighted Analyses

The results of the multivariable and propensity-weighted analyses are summarized in Table 3. Adjusted effect estimates from the multivariable models are illustrated in Figure 3. Following adjustment for recipient age, physiological MELD score, donor age, and ischemia time, the Pringle maneuver remained independently associated with several favorable perioperative and postoperative outcomes. Recipient hepatectomy duration was significantly shorter in the Pringle group (time ratio, 0.76; 95% CI, 0.67–0.87; *p* < 0.001), while FFP transfusion requirements were significantly reduced (IRR, 0.60; 95% CI, 0.40–0.91; *p* = 0.015). The composite endpoint of total blood product transfusion was likewise significantly reduced (IRR, 0.61; 95% CI, 0.40–0.92; *p* = 0.020). In addition, the odds of postoperative bleeding (OR, 0.04; 95% CI, 0.01–0.25; *p* < 0.001), biliary leak (OR, 0.13; 95% CI, 0.02–0.63; *p* = 0.010), and early allograft dysfunction (OR, 0.14; 95% CI, 0.04–0.58; *p* = 0.006) remained significantly lower following multivariable adjustment. Although PRBC transfusion requirements remained numerically lower in the Pringle group, the association did not reach statistical significance after adjustment (IRR, 0.70; 95% CI, 0.43–1.12; *p* = 0.139).

Inverse probability of treatment weighting (IPTW) substantially improved baseline covariate balance, reducing the maximum absolute standardized mean difference from approximately 0.51 before weighting to 0.16 after weighting (Figure 4). Following IPTW adjustment, the weighted analyses yielded results generally consistent with those from the multivariable regression models. Significant associations persisted for recipient hepatectomy duration (time ratio, 0.76; 95% CI, 0.68–0.85; *p* < 0.001), FFP transfusion (IRR, 0.59; 95% CI, 0.43–0.82; *p* = 0.002), total blood product transfusion (IRR, 0.65; 95% CI, 0.42–0.99; *p* = 0.046), postoperative bleeding (OR, 0.05; 95% CI, 0.01–0.23; *p* < 0.001), biliary leakage (OR, 0.11; 95% CI, 0.03–0.51; *p* = 0.005), and early allograft dysfunction (OR, 0.30; 95% CI, 0.09–0.96; *p* = 0.043). Neither the adjusted Cox regression analysis (HR, 0.36; 95% CI, 0.13–1.06; *p* = 0.064) nor the propensity-weighted analysis (OR, 0.46; 95% CI, 0.14–1.48; *p* = 0.192) demonstrated a statistically significant association between the Pringle maneuver and mortality. As illustrated in Figure 3, all statistically significant adjusted effect estimates favored the Pringle maneuver, whereas the confidence intervals for PRBC transfusion and mortality crossed the null value (effect estimate = 1).

### 3.4. Overall Survival

Overall survival was evaluated using Kaplan–Meier analysis and is presented in Figure 5. During a median follow-up of 28.6 months, 20 deaths occurred, including 10 in the Pringle group and 10 in the No-Pringle group. One-year overall survival was 86.5% in the Pringle group and 88.7% in the No-Pringle group. Kaplan–Meier analysis demonstrated no significant difference in overall survival between the two groups (log-rank *p* = 0.885).

Consistent with the unadjusted survival analysis, multivariable Cox regression showed no statistically significant association between the Pringle maneuver and mortality (HR, 0.36; 95% CI, 0.13–1.06; *p* = 0.064). Similarly, propensity-weighted analysis demonstrated no significant difference in mortality between the two groups (OR, 0.46; 95% CI, 0.14–1.48; *p* = 0.192) (Table 3).

### 3.5. Temporal Trends Within the Study Periods

To assess whether the improvement in perioperative outcomes could reflect a gradual gain in institutional experience rather than the introduction of the Pringle maneuver, trends over calendar time were examined within each period (Supplementary Figure S1). During the three years preceding the introduction of the maneuver (2020–2022), recipient hepatectomy duration showed no trend with the date of transplantation (Spearman ρ = −0.01, *p* = 0.954; annual medians of 173, 190, and 176 min in 2020, 2021, and 2022, respectively), and both hepatectomy duration (177 vs. 176 min, *p* = 0.697) and total blood product use (8 vs. 7 units, *p* = 0.283) were similar in the first and second halves of that period. PRBC use declined modestly across the No-Pringle period (ρ = −0.27, *p* = 0.040), whereas FFP (ρ = −0.10, *p* = 0.433) and total blood product use (ρ = −0.21, *p* = 0.114) did not. Within the Pringle period (2023–2026), a further decrease over time was observed for hepatectomy duration (ρ = −0.28, *p* = 0.012; annual medians of 151, 126.5, 120, and 105.5 min in 2023, 2024, 2025, and 2026, respectively) and for total blood product use (ρ = −0.34, *p* = 0.002), with lower values in the second than in the first half of the period (hepatectomy duration 120 vs. 149 min, *p* = 0.041; total blood products 3 vs. 6 units, *p* = 0.010).

## 4. Discussion

In accordance with our study hypothesis, routine application of the Pringle maneuver was associated with improved operative efficiency and more favorable early postoperative outcomes. Although recipients in the Pringle group required fewer PRBC transfusions in the unadjusted analysis, this association did not remain statistically significant after multivariable adjustment. In contrast, routine Pringle maneuver remained independently associated with a lower total blood product requirement, reduced FFP transfusion, shorter recipient hepatectomy, lower postoperative bleeding, fewer biliary leaks, and a lower incidence of early allograft dysfunction. Collectively, these findings suggest that improved intraoperative hemostasis may enhance early graft function without adversely affecting patient survival.

Recipient hepatectomy is a complex and challenging aspect of the liver transplant operation. Advanced cirrhosis, severe portal hypertension, extensive portosystemic collateralization, and coagulopathy substantially increase the risk of major intraoperative bleeding during recipient hepatectomy [27,28]. Increased bleeding during the operation compromises exposure of critical retrohepatic and hilar structures and often leads to the need for intraoperative blood transfusion, prolonged dissection, and multiple hemostatic procedures [27,28,29]. Increased use of hepatic inflow occlusion during liver surgery has improved hemostasis and has been shown to improve dissection of the hepatic parenchyma and the hilar region [12,13,14,15,16,17,18,19,20]. Our findings suggest that improved intraoperative hemostasis may facilitate recipient hepatectomy, thereby reducing operative duration and postoperative bleeding. Beyond these measurable outcomes, the operating surgeons observed that routine inflow occlusion facilitated the recipient hepatectomy, making this stage more suitable for the graded training and mentoring of transplant fellows.

While the Pringle maneuver has received considerable attention in the context of liver resection, the same cannot be said for its application in liver transplantation. Most of what we know comes from reports that are limited to the living-donor transplantation context, where operative conditions, hemodynamic physiology, and ischemia–reperfusion injury differ substantially from those encountered during deceased-donor liver transplantation [22,23,24]. Recent studies in liver transplantation have reported that temporary hepatic inflow occlusion reduces intraoperative bleeding and transfusion requirements without increasing ischemia–reperfusion injury or postoperative complications [30,31,32]. However, those studies have mostly focused on the surgical aspect. In contrast to those studies, our study contributes to the existing body of knowledge by showing that a significant reduction in EAD, a clinically meaningful outcome, occurs alongside improved surgical efficiency. This outcome has been largely overlooked in the prior studies.

The decline in EAD observed in our study is of particular clinical importance. EAD is a well-established predictor of early postoperative morbidity, prolonged hospitalization, impaired graft function, and reduced long-term graft survival [29,33]. The mechanisms linking hepatic inflow occlusion during recipient hepatectomy to reduced EAD are likely multifactorial [22,34]. Improved visualization of the operative field may facilitate more meticulous hilar dissection and vascular control while minimizing diffuse venous bleeding from portal hypertensive collateral vessels [28]. Inflow occlusion also decompresses the congested liver, producing an appreciable reduction in hepatic volume and turgor. The smaller, softer liver can be rotated medially with greater ease, improving exposure of the retrohepatic space and permitting safer division of the short hepatic veins and more controlled dissection of the inferior vena cava. This effect is attenuated in recipients with markedly enlarged livers, in whom the absolute size of the organ continues to limit access to the hilum. Reduced intraoperative bleeding and transfusion requirements may decrease transfusion-related inflammatory responses, coagulopathy, and hemodynamic instability, thereby reducing the cumulative physiological stress imposed on the newly transplanted graft [35]. Furthermore, shorter recipient hepatectomy may limit prolonged operative exposure and facilitate a more controlled transition to graft implantation. Although these mechanisms remain speculative, they provide biologically plausible explanations for the observed association between routine hepatic inflow occlusion and reduced EAD [27,35].

An important consideration, however, is that donor age and warm ischemia time differed significantly between the study groups. Both variables are recognized determinants of early graft function and have been associated with the risk of EAD. Accordingly, the lower incidence of EAD observed in the Pringle group may have been influenced, at least in part, by differences in donor characteristics. To address this potential confounding, donor age and ischemia time were incorporated into the multivariable regression models, and baseline differences were further balanced using inverse probability of treatment weighting. Notably, the association between routine Pringle maneuver and reduced EAD remained statistically significant in both adjusted analyses, suggesting that differences in donor characteristics alone are unlikely to fully explain the observed findings. Nevertheless, residual confounding inherent to this retrospective study design cannot be completely excluded.

In a multivariable context, the significance of PRBC transfusion diminished, while the association between the decreasing rate of FFP transfusion and the Pringle maneuver remained significant. There are several possible reasons for this observation. The transfusion of PRBCs depends on multiple factors, including the patient’s baseline hemoglobin concentration, intraoperative hemodynamic management, transfusion thresholds, and various factors related to the anesthetist. In addition, several recipients received PRBC transfusions at the beginning of the transplantation procedure because of pre-existing anemia rather than active intraoperative bleeding. Consequently, PRBC transfusion alone may not accurately reflect the true hemostatic effect of the Pringle maneuver. Conversely, the transfusion of FFP may depend largely on the severity of bleeding during the operation and the degree of perturbation of the coagulation system [36,37]. For this reason, transfusion of FFP may be a more accurate indicator of improved surgical hemostasis in this case [36]. When PRBC and FFP were combined into a single measure of total transfusion burden, the association with the Pringle maneuver remained significant after both multivariable adjustment and propensity weighting, indicating that the overall reduction in transfusion requirement is not attributable to the FFP component alone. No major differences in overall survival between groups were found. Only 20 recipients died during the study. Recipients in the Pringle group had a shorter follow-up duration because routine implementation of the Pringle maneuver occurred later in the study period. Consequently, the study was underpowered to detect moderate differences in long-term survival. However, the absence of an adverse survival outcome indicates that this maneuver was safe. Larger studies with longer follow-up are needed to determine whether early graft function improves without adversely affecting long-term outcomes.

The principle of reducing hepatic inflow before mobilization of the native liver is not new. Preservation of the retrohepatic vena cava (piggyback technique) [38] changed the sequence of recipient hepatectomy, and several strategies have been described to decompress the portal system before the liver is dissected off the cava, including early (“hilum-first”) control and division of the portal vein with a temporary end-to-side portocaval shunt [39,40]. In a randomized trial, a temporary portocaval shunt improved hemodynamic stability during the anhepatic phase and reduced transfusion requirements [40], and a meta-analysis of six studies confirmed lower PRBC use, lower postoperative transaminase levels, and better renal function with this approach [41]. The intermittent Pringle maneuver differs from these techniques in three respects: it is reversible and can be applied and released repeatedly according to the hemodynamic response; it requires no additional anastomosis and no early division of the portal vein, so that the hilar structures are preserved until the liver has been fully mobilized; and it interrupts arterial as well as portal inflow, which decompresses the liver and the hilar collaterals more completely. Its disadvantage is that, unlike a portocaval shunt, it does not relieve splanchnic congestion during the period of occlusion, which is why our protocol limits each occlusion to 10–15 min with reperfusion intervals and bases the number of cycles on the hemodynamic status and the appearance of the small bowel. Our study did not compare the Pringle maneuver with a hilum-first approach or a temporary portocaval shunt and cannot establish superiority over these techniques; in centers where the classic hepatectomy with excision of the retrohepatic cava is still practiced, inflow occlusion could in principle serve the same purpose during mobilization, but this was not examined here. A prospective comparison of these inflow-control strategies within a caval-preserving hepatectomy would be of considerable interest.

An alternative, non-surgical explanation for part of the benefit deserves consideration. Intermittent inflow occlusion imposes a stepwise rather than an abrupt reduction in venous return, and each reperfusion interval gives the anesthesia team time to restore preload and to titrate vasopressors gradually; it is therefore possible that some of the reduction in transfusion and in early graft dysfunction reflects more controlled resuscitation rather than a direct hemostatic effect. Hemodynamic instability after graft reperfusion (post-reperfusion syndrome) is a recognized contributor to coagulopathy, transfusion requirement, and early graft injury [42,43], and although the Pringle maneuver is applied before the anhepatic phase, a recipient who reaches reperfusion after less blood loss and with a smaller cumulative transfusion burden may tolerate reperfusion better. We were unable to examine these mechanisms because intravenous fluid volumes, vasopressor and inotrope doses, hemodynamic data at reperfusion, and the occurrence of post-reperfusion syndrome were not captured in our database, and coagulation was monitored mainly by clinical assessment and conventional laboratory tests in both periods, with only occasional, non-routine use of thromboelastography, so that a change in monitoring strategy between the periods is unlikely. Viscoelastic testing has been shown to reduce plasma transfusion during liver transplantation [44], and because FFP was the transfusion component most clearly reduced in our study, differences in the use of other hemostatic agents could contribute to the observed effect; the stability of the anesthetic protocol and of the monitoring strategy across the study period makes a systematic difference unlikely, but it cannot be excluded. Prospective collection of hemodynamic, fluid, and viscoelastic data during recipient hepatectomy with and without inflow occlusion is needed to disentangle these mechanisms.

The lower incidence of biliary leak in the Pringle group also requires cautious interpretation. Anastomotic bile leak after liver transplantation is determined mainly by technical factors, by the vascular supply of the donor and recipient bile ducts, and by donor and graft quality, with older donor age, marginal grafts, and prolonged ischemia recognized as risk factors [45]. A direct protective effect of hepatic inflow occlusion on the biliary anastomosis is not biologically obvious, because the maneuver is applied to the native liver before the anhepatic phase. More plausible pathways are indirect: a shorter and less bloody hepatectomy preserves the periductal tissue and the arterial supply of the recipient duct, avoids extensive hemostatic maneuvers in the hilum, and allows the anastomosis to be performed in a drier field with less coagulopathy and tissue edema, while lower transfusion requirements and greater hemodynamic stability may reduce ischemic injury to the biliary epithelium of the graft. Era-related factors cannot be separated from these mechanisms. The surgeons performing the biliary reconstruction did not change during the study period; however, in some recipients of the No-Pringle group the duct-to-duct anastomosis was performed over an internal plastic stent, whereas stents were not used in the Pringle group. The benefit of internal stenting in duct-to-duct reconstruction is uncertain: a meta-analysis of 17 studies including 2623 patients found no reduction in bile leak or stricture with stenting, and stented patients more often required repeated endoscopic interventions [46], and in a large single-center series internal stenting did not prevent anastomotic leaks and was associated with more post-transplant biliary interventions [47]. A contribution of the stent to the higher leak rate in the No-Pringle group therefore cannot be excluded. Together with the cumulative experience of the wider transplant team, the younger donors in the Pringle group, and the small number of events (nine leaks in total), this limits inference. Although the association persisted after adjustment for donor age and ischemia time and after propensity weighting, we regard the reduction in biliary leak as a secondary, hypothesis-generating finding that should be confirmed in larger series.

Several limitations should be considered when interpreting these findings. First, the retrospective, single-center design precludes definitive causal inference. Second, the use of a historical control group introduces the possibility of era-related confounding, as improvements in team experience, perioperative management, anesthetic techniques, and postoperative care over time may have contributed to the observed differences. To minimize this potential bias, we adjusted for clinically relevant baseline characteristics using multivariable regression and additionally performed inverse probability of treatment weighting, both of which yielded consistent results. Moreover, the same two experienced surgeons performed all procedures in both periods, so an individual surgical learning curve is an unlikely explanation for the differences between the periods. In addition, the within-period analysis showed no improvement in hepatectomy duration or total transfusion across the three years preceding the introduction of the maneuver, which argues against a gradual learning curve as the sole explanation for the step change observed in 2023; however, outcomes continued to improve within the Pringle period, consistent with a learning curve for the maneuver itself, with concurrent refinements in perioperative care, or with both, so that era effects cannot be fully separated from the effect of the intervention [48]. The recipient and donor characteristics that were available, including the indication for transplantation, markers of hepatic decompensation, and extended-criteria donor status, did not differ between the periods, and the Pringle group, if anything, included sicker recipients; nevertheless, several potentially relevant variables, including intraoperative fluid and vasopressor administration, post-reperfusion hemodynamics, and graft histology, were not recorded and could not be accounted for. Nevertheless, residual confounding from unmeasured temporal factors cannot be excluded. Third, donor age and warm ischemia time differed between groups despite statistical adjustment, and these differences may have influenced early graft outcomes. In addition, an internal biliary stent was used only in some recipients of the No-Pringle group, which may have influenced the biliary outcomes. Fourth, the absolute numbers of PRBC and FFP units transfused were relatively small in both groups, and the between-group differences, although statistically significant, were modest in absolute terms; together with the historical-control design and the residual imbalance in donor age and warm ischemia time, this means that less weight should be placed on any individual adjusted estimate than on the consistency of the direction of effect across outcomes and analytical approaches. Finally, the relatively modest sample size and limited number of events reduced the statistical power to detect differences in less frequent outcomes, particularly long-term survival. Accordingly, these findings should be considered hypothesis-generating and warrant confirmation in prospective multicenter studies.

In conclusion, routine intermittent application of the Pringle maneuver during recipient hepatectomy was associated with improved operative efficiency, reduced transfusion requirements, and a significantly lower incidence of early allograft dysfunction after adult orthotopic liver transplantation. These findings support routine intermittent hepatic inflow occlusion as a simple surgical adjunct that may improve early transplant outcomes, although confirmation in prospective multicenter studies is warranted.

## Figures and Tables

**Figure 1:**
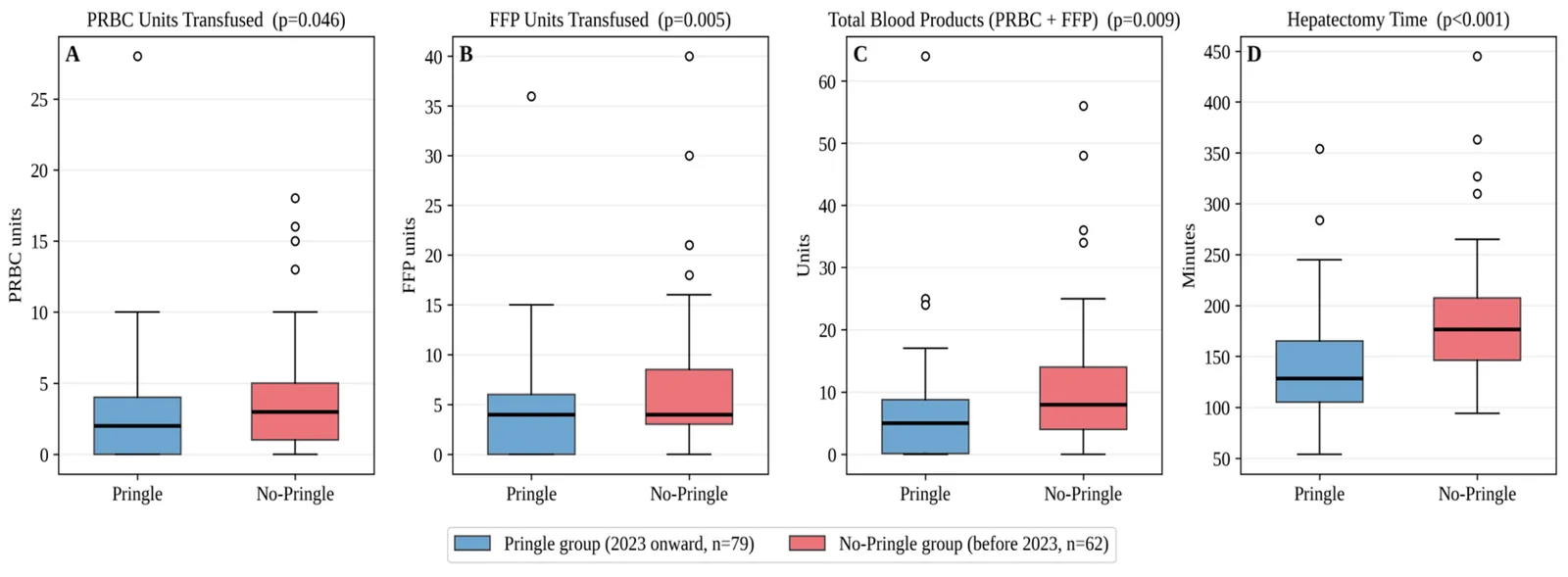
Comparison of perioperative outcomes between the Pringle and No-Pringle groups. Box-and-whisker plots comparing (**A**) packed red blood cell (PRBC) transfusion, (**B**) fresh frozen plasma (FFP) transfusion, (**C**) total blood product transfusion (PRBC + FFP), and (**D**) recipient hepatectomy duration between recipients undergoing liver transplantation with routine application of the Pringle maneuver (Pringle group) and those without hepatic inflow occlusion (No-Pringle group). Boxes represent the interquartile range {IQR}; the central horizontal line indicates the median; whiskers extend to 1.5 × IQR; and individual circles represent outliers. *p*-values were calculated using the Mann–Whitney U test.

**Figure 2:**
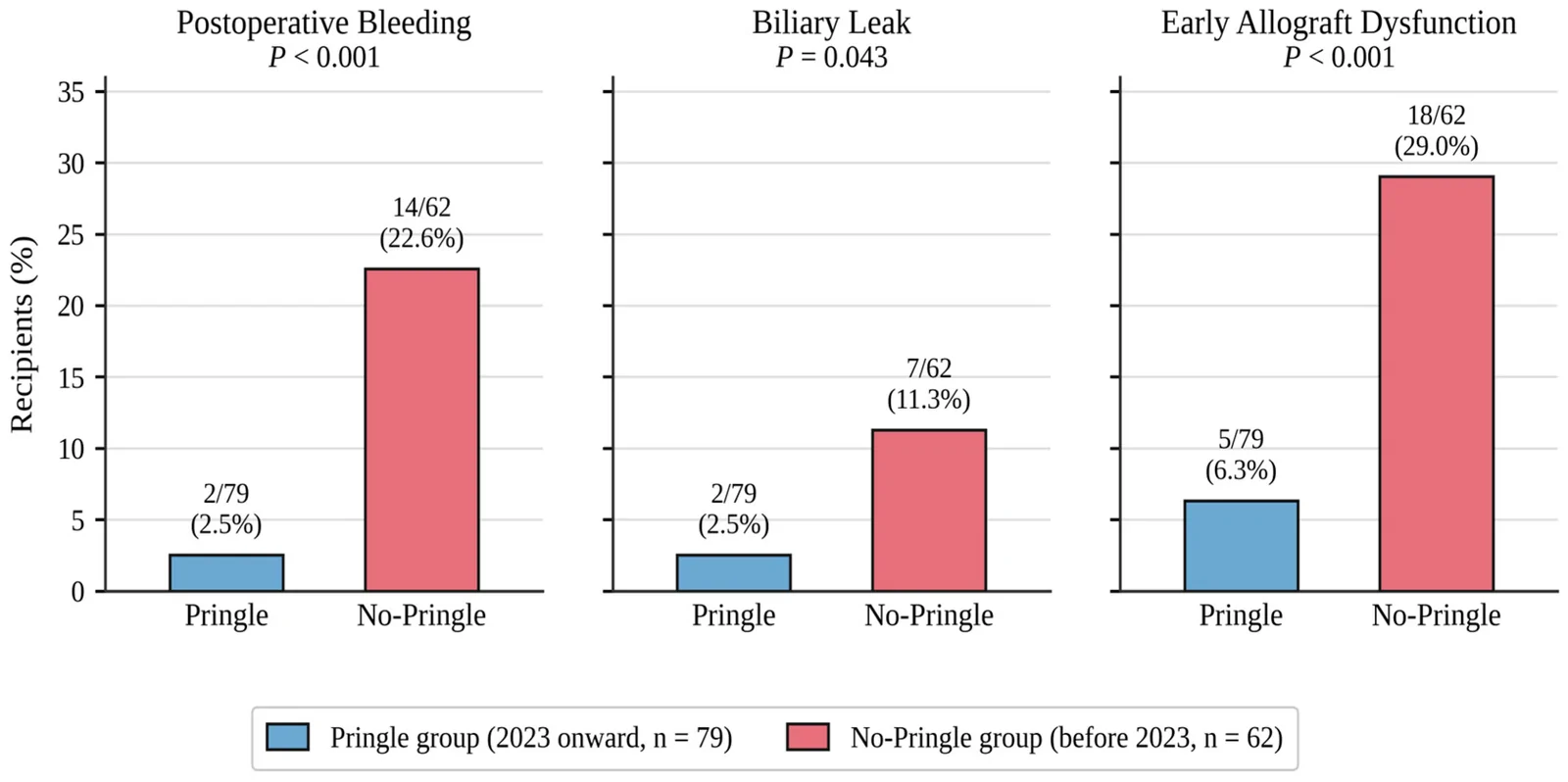
Early postoperative complications according to study group. Bars represent the proportion of recipients who developed postoperative bleeding, biliary leak, and early allograft dysfunction after liver transplantation in the Pringle and No-Pringle groups. Numbers above each bar indicate the number of events over the number of recipients at risk and the corresponding percentage. *p* values were calculated using the χ^2^ test, or Fisher’s exact test when any expected cell count was less than 5.

**Figure 3:**
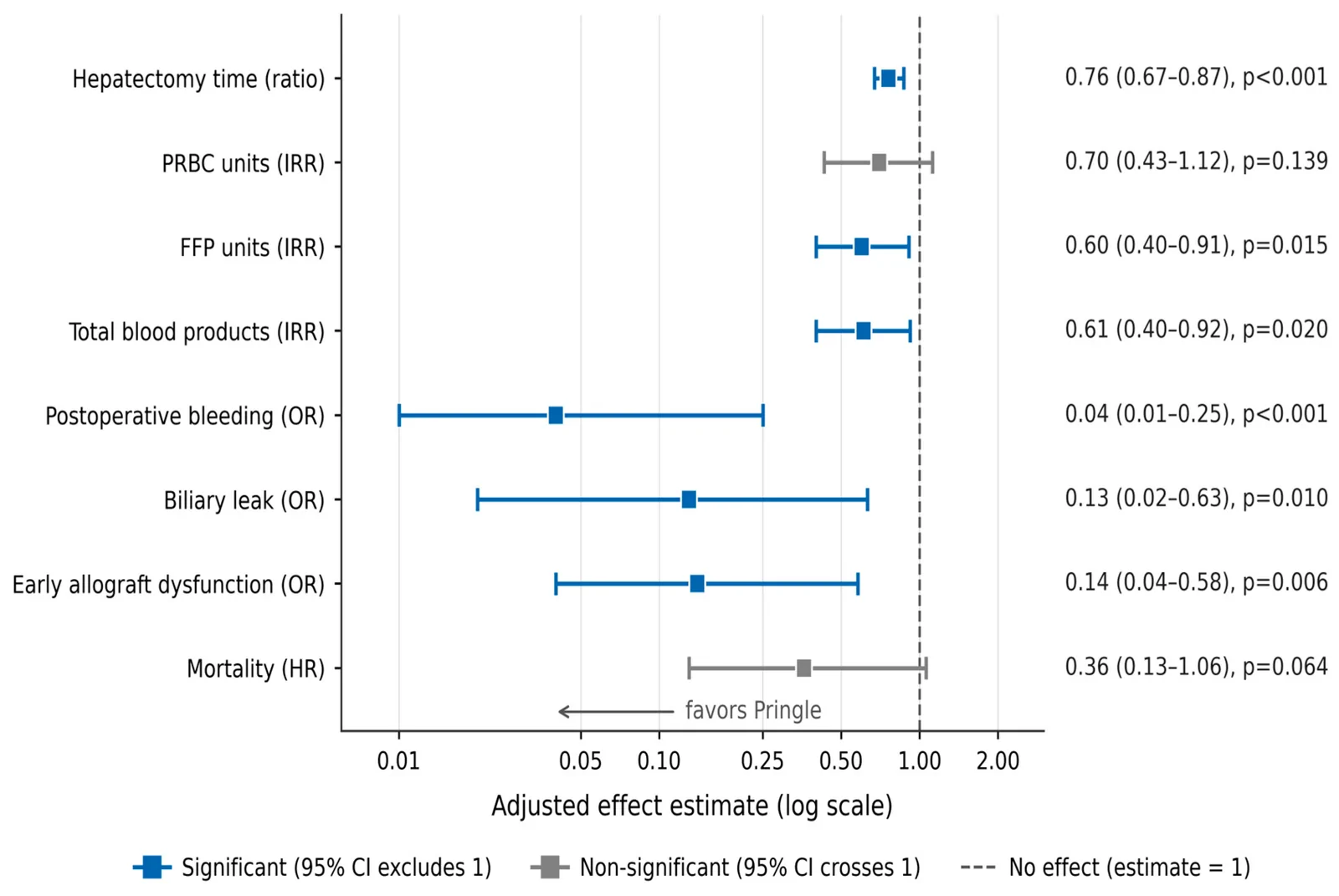
Forest plot of adjusted associations between the Pringle maneuver and perioperative and postoperative outcomes. Adjusted effect estimates are presented as time ratios, incidence rate ratios (IRRs), odds ratios (ORs), or hazard ratios (HRs), with 95% confidence intervals. Values below 1 favor the Pringle maneuver, and the vertical dashed line indicates the null value (effect estimate = 1).

**Figure 4:**
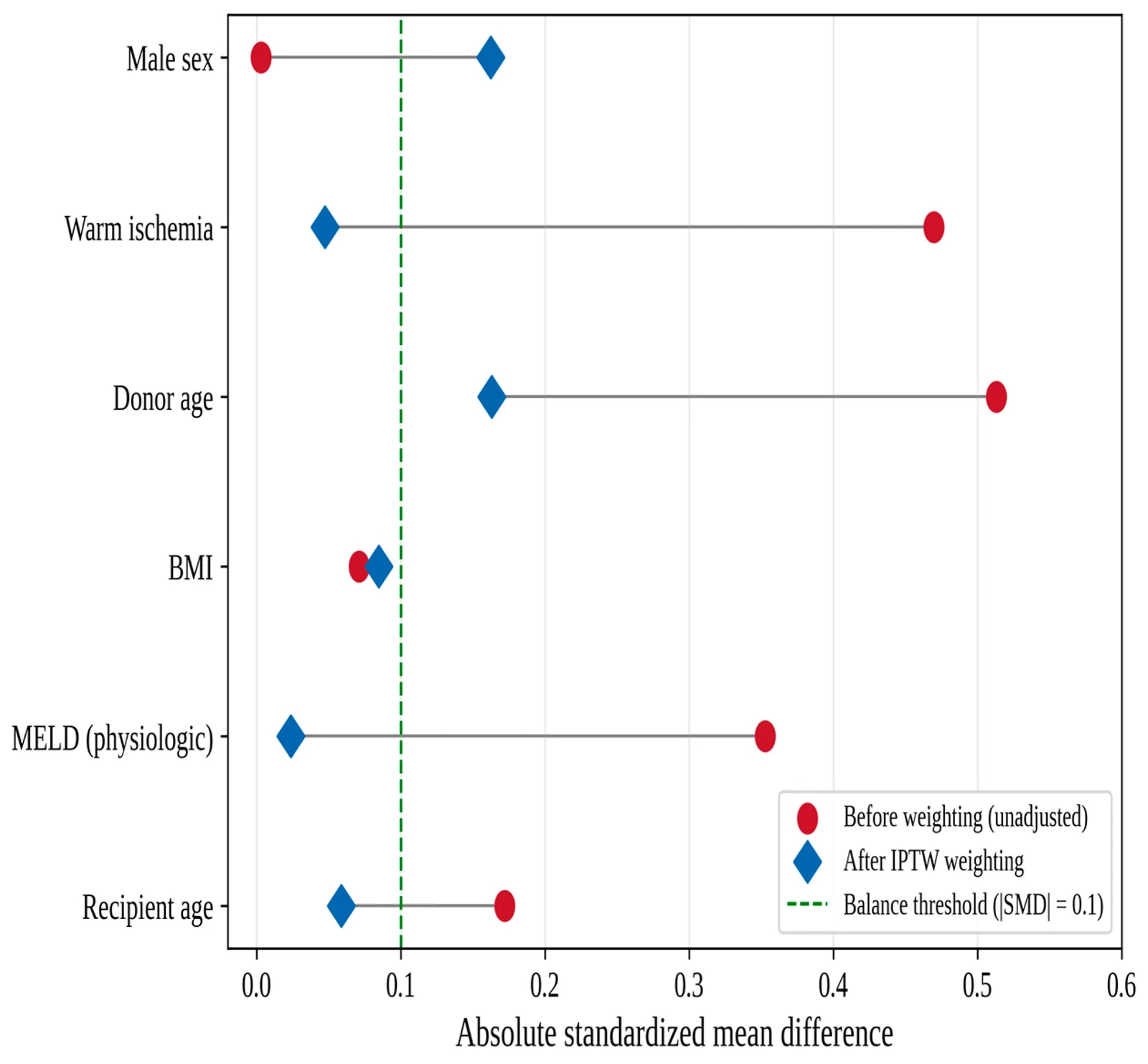
Covariate balance before and after propensity-score (IPTW) weighting. Absolute standardized mean differences for each covariate before (circles) and after (diamonds) inverse probability of treatment weighting. The dashed vertical line indicates the conventional standardized mean difference threshold of 0.10 for adequate covariate balance.

**Figure 5:**
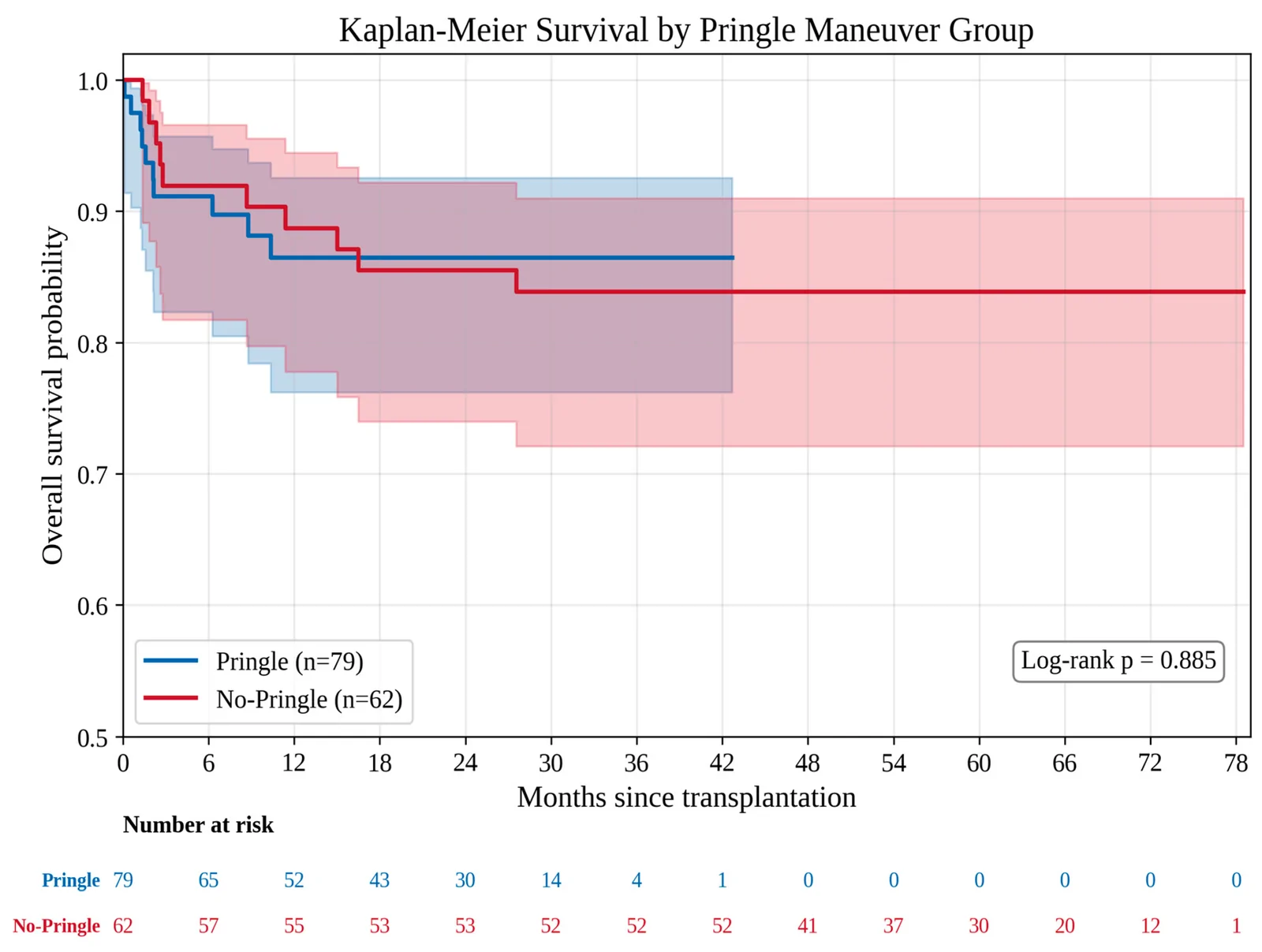
Kaplan–Meier overall survival according to study group. Kaplan–Meier curves comparing overall survival after adult liver transplantation between recipients who underwent transplantation with routine use of the Pringle maneuver (Pringle group) and those without hepatic inflow occlusion (No-Pringle group). Shaded areas represent 95% confidence intervals. The table below the graph shows the number of patients at risk at each time point. Survival curves were compared using the log-rank test.

**Table 1:** Baseline characteristics by group.

Variable	Pringle (n = 79)	No-Pringle (n = 62)	*p* Value
Recipient age, years	62 {53–68}	58 {46–66}	0.055
Male sex	47 (59.5)	39 (62.9)	0.812
Primary indication for transplantation			0.542
MASLD	33 (43.4)	21 (36.2)	
Viral hepatitis (HBV or HCV)	9 (11.8)	7 (12.1)	
Autoimmune or cholestatic	9 (11.8)	12 (20.7)	
Alcohol-related	6 (7.9)	2 (3.4)	
Other	19 (25.0)	16 (27.6)	
Hepatocellular carcinoma	23 (30.3)	11 (19.0)	0.197
Ascites	47 (60.3)	32 (54.2)	0.595
Hepatic encephalopathy, any grade	37 (47.4)	18 (30.5)	0.068
Pre-transplant location			0.200
Home	46 (59.0)	33 (55.9)	
Hospital ward	19 (24.4)	21 (35.6)	
Intensive care unit	13 (16.7)	5 (8.5)	
MELD, physiologic (laboratory), at transplantation	26 {23–31}	24 {22–27}	0.065
BMI, kg/m^2^	26.9 {24.2–31.4}	26.8 {24.0–31.5}	0.958
Donor age, years	53 {40–71}	65 {56–74}	0.007
Extended-criteria donor	39 (50.0)	36 (61.0)	0.267
Cold ischemia time, min	303 {260–352}	315 {262–350}	0.587
Warm ischemia time, min	33 {29–38}	35 {31–40}	0.028

Data are median {interquartile range} unless otherwise stated. MELD, Model for End-stage Liver Disease. The physiologic MELD is the laboratory MELD calculated immediately before transplantation. Continuous variables were compared using the Mann–Whitney U test and categorical variables using the χ^2^ test, or Fisher’s exact test when any expected cell count was less than 5. All tests were two-sided; *p* < 0.05 was considered statistically significant. Data on the indication for transplantation were available for 76 recipients in the Pringle group and 58 in the No-Pringle group; data on ascites, encephalopathy, pre-transplant location, and extended-criteria donor status were available for 78 and 59 recipients, respectively, and percentages refer to recipients with available data. Other indications comprise acute or acute-on-chronic liver failure (n = 5), inherited metabolic disease (n = 9), Budd–Chiari syndrome (n = 3), and cryptogenic or other causes (n = 18). HBV, hepatitis B virus; HCV, hepatitis C virus; MASLD, metabolic dysfunction-associated steatotic liver disease.

**Table 2:** Unadjusted perioperative and early postoperative outcomes according to study group.

Outcome	Pringle (n = 79)	No-Pringle (n = 62)	*p* Value
PRBC transfusion, units	2 {0–4}	3 {1–5}	0.046
FFP transfusion, units	4 {0–6}	4 {3–8}	0.005
Total blood products, units	5 {0–9}	8 {4–14}	0.009
No intraoperative transfusion	20 (25.3)	6 (9.7)	0.031
Recipient hepatectomy time, min	129 {105–165}	177 {146–208}	<0.001
Postoperative bleeding	2 (2.5)	14 (22.6)	<0.001
Biliary leak	2 (2.5)	7 (11.3)	0.043
Postoperative ileus	1 (1.3)	1 (1.6)	1.000
Early allograft dysfunction	5 (6.3)	18 (29.0)	<0.001
Death during follow-up	10 (12.7)	10 (16.1)	0.731

Continuous variables are presented as median (interquartile range {IQR}), and categorical variables as number (percentage). PRBC transfusion was used as a surrogate measure of intraoperative blood loss. EAD was defined according to the criteria of Olthoff et al. as the presence of one or more of the following: serum bilirubin ≥ 10 mg/dL or INR ≥ 1.6 on postoperative day 7, or AST or ALT > 2000 IU/L within the first 7 postoperative days. PRBC, packed red blood cells; FFP, fresh frozen plasma; EAD, early allograft dysfunction. Continuous variables were compared using the Mann–Whitney U test and categorical variables using the χ^2^ test, or Fisher’s exact test when any expected cell count was less than 5. All tests were two-sided; *p* < 0.05 was considered statistically significant.

**Table 3:** Adjusted and propensity-weighted effect of the Pringle maneuver on each outcome.

Outcome (Measure)	Adjusted (95% CI)	*p* Value	IPTW (95% CI)	*p* Value
Hepatectomy time (time ratio)	0.76 (0.67–0.87)	<0.001	0.76 (0.68–0.85)	<0.001
PRBC units (IRR)	0.70 (0.43–1.12)	0.139	0.77 (0.58–1.03)	0.082
FFP units (IRR)	0.60 (0.40–0.91)	0.015	0.59 (0.43–0.82)	0.002
Total blood products (IRR)	0.61 (0.40–0.92)	0.020	0.65 (0.42–0.99)	0.046
Postoperative bleeding (OR)	0.04 (0.01–0.25)	<0.001	0.05 (0.01–0.23)	<0.001
Biliary leak (OR)	0.13 (0.02–0.63)	0.010	0.11 (0.03–0.51)	0.005
Early allograft dysfunction (OR)	0.14 (0.04–0.58)	0.006	0.30 (0.09–0.96)	0.043
Mortality (HR/OR)	0.36 (0.13–1.06)	0.064	0.46 (0.14–1.48)	0.192

Effect estimates are for the Pringle group relative to the No-Pringle group; values below 1.0 favor the Pringle maneuver. Multivariable models were adjusted for recipient age, physiologic MELD score, donor age, and warm ischemia time; in the early allograft dysfunction model, cold ischemia time was substituted for warm ischemia time. The adjusted estimate for biliary leak was obtained by Firth penalized maximum likelihood because of the small number of events (n = 9), with the 95% CI and *p*-value derived from the penalized profile likelihood; all other logistic models were fitted by ordinary maximum likelihood. The adjusted mortality estimate is a Cox proportional hazards ratio; the IPTW mortality estimate is a weighted odds ratio. All tests were two-sided; *p* < 0.05 was considered statistically significant. CI, confidence interval; HR, hazard ratio; IPTW, inverse probability of treatment weighting; IRR, incidence rate ratio; OR, odds ratio; PRBC, packed red blood cells; FFP, fresh frozen plasma.

## Data Availability

The datasets generated and/or analyzed during the current study are available from the corresponding author upon reasonable request.

## Supplementary Materials

The following supporting information can be downloaded at: https://www.mdpi.com/article/10.3390/jcm15207939/s1, Figure S1. Recipient hepatectomy duration and total intraoperative blood product use according to the date of transplantation. Figure S2. Distribution of intraoperative fresh frozen plasma (FFP) transfusion in the Pringle (n = 78) and No-Pringle (n = 59) groups.
