# Supplementary material for: Routine Pringle Maneuver During Recipient Hepatectomy Is Associated with Improved Perioperative and Early Graft Outcomes After Adult Liver Transplantation
Source: J Clin Med. 2026 Oct 14;15(20):7939. doi: 10.3390/jcm15207939 (PMC13705742; doi:10.3390/jcm15207939)
Supplement: Supplementary file 1 [file jcm-15-07939-s001.zip › jcm-4534739-supplementary.pdf]

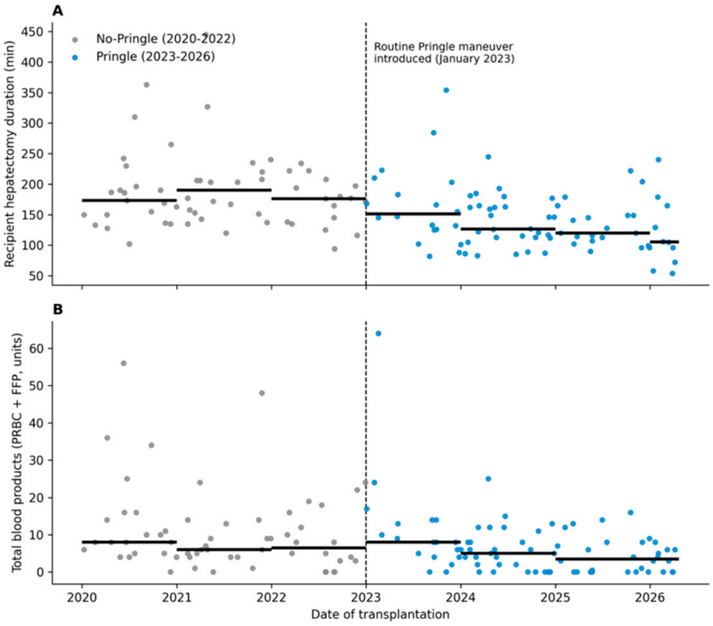

Supplementary Figure S1. Recipient hepatectomy duration and total intraoperative blood product use according to the date of transplantation. Each circle represents one recipient; horizontal bars indicate the annual median. The dashed vertical line marks the routine introduction of the intermittent Pringle maneuver in January 2023. Spearman rank correlations with the date of transplantation were calculated separately within the No-Pringle (2020-2022) and Pringle (2023-2026) periods.

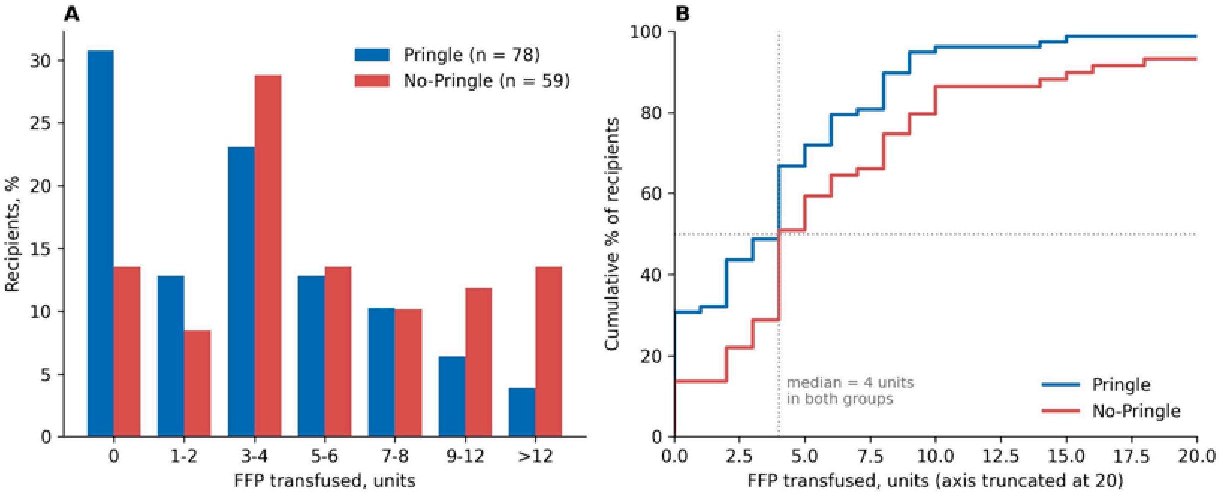

Supplementary Figure S2. Distribution of intraoperative fresh frozen plasma (FFP) transfusion in the Pringle (n = 78) and No-Pringle (n = 59) groups. (A) Proportion of recipients according to the number of FFP units transfused. (B) Cumulative distribution of FFP units; the dotted lines mark the median of 4 units, which was identical in the two groups, whereas the distribution in the Pringle group is shifted towards lower values (no FFP in 30.8% vs. 13.6%; mean 4.1 vs. 6.9 units; 75th and 90th percentiles 6 and 8 vs. 8.5 and 15 units; Hodges-Lehmann difference -2 units, 95% CI -4 to 0; Mann-Whitney P = 0.005). The horizontal axis in panel B is truncated at 20 units (one Pringle-group recipient

and four No-Pringle recipients received more than 20 units). FFP data were missing for one Pringle-group recipient and three No-Pringle recipients.
